# COVID-19 Pandemic and Its Impact on the Quality of Women’s Sexual Life: A Systematic Review

**DOI:** 10.3390/healthcare11020185

**Published:** 2023-01-07

**Authors:** Anastasia Voutskidou, Giannoula Kirkou, Maria Dagla, Eirini Orovou, Angeliki Sarella, Ermioni Palaska, Maria Iliadou, Evangelia Antoniou

**Affiliations:** Department of Midwifery, University of West Attica, Agioy Spyridonos 28, 12243 Egaleo, Greece

**Keywords:** COVID-19, SARS-CoV-2, Coronavirus, COVID-19 pandemic, female sexual function, female sexual behavior, female sexual health

## Abstract

The COVID-19 pandemic has had far-reaching effects, including onphysical and mental health and wellbeing. The aim of this study was to investigate the effect of the COVID-19 pandemic on women’s health, especially on women’s sexual life includingdesire, arousal, orgasm and satisfaction. The initial research in PubMed/Medline, Google Scholar and Scopus yielded 573 articles from Europe (Portugal, Poland, Italy, Greece), America (U.S, Brazil) and Africa (Egypt), of which 14 met the inclusion criteria and were included in the review. Results from the studies suggest that the pandemic negatively affected sexual functioning and satisfaction, while it increased sexual distress, sexual avoidance and solitary sexual approach behaviors. The desire to have children decreased during the pandemic, but so didthe use of contraception. There is conflicting evidence as to whether or not the pandemic has had an impact on sexual desire and frequency among women. However, some major factors associated with sexual life were psychological factors and working status. It is well documented that the pandemic has had a great psychological impact. Therefore, both of these factors are expected to significantly affect women’s sexual life.

## 1. Introduction

Due to the COVID-19 pandemic, several non-pharmaceutical interventions had to be utilized by governments from all over the world in order to reduce SARS-CoV-2 transmission and keep the numbers of patients in hospitals as manageable as possible. The main social distancing methods included stay-at-home orders, remote working when possible, external activities only being allowed with granted permission, reduction in the size of public and private gatherings, travel restrictions, closure of high-exposure businesses, as well as shops, schools and universities [1,2]. As citizens spent their days almost exclusively at their homes, any physical contact between families, friends and couples who did not live together was prevented. These measures directly or indirectly disrupted the lives of people as well as health care delivery systems in many countries, as has been seen in other pandemics or disasters. In particular, quarantine and isolation have profound psychological and social effects such as increased stress, anxiety, fear, loneliness, depression, other psychiatric disorders, substance use and suicide [3]. As a consequence, couples’ sex lives were affected too. Women and men were wondering how to handle marital relationships in quarantine and whether COVID-19 could be transmitted through sexual intercourse. Longer contact between couples during home quarantine intensified negative feelings and increased marital conflicts [4].

Female sexual function involves the cycle of sexual response which includes three main phases: desire, excitement and orgasm. According to the three-phase model by Helen Singer Kaplan, physiological, psychological, hormonal and also social factors affect sexual desire and libido, and thereafter orgasm [5].Therefore, normal female sexual function is characterized by the absence of difficulty in transitioning through these three stages, the absence of pain and finally the achievement of sexual satisfaction. Sexual dysfunction refers to transient difficulties of the individual with being satisfied by sexual activity for at least 6 months or more. According to the earliest version of the American Psychiatric Association’s Diagnostic and Statistical Manual of Mental Disorders (DSM-5), most sexual dysfunctions are a result of both biological and psychological factors such as medical factors, the individual’s vulnerability, relationship, partner, and cultural or religious factors. Specifically for females, sexual desire and arousal disorders refer to female sexual interest/arousal disorder [6]. Other measures used to assess women’s sexual life are sexual satisfaction and sexual distress. Sexual satisfaction has been described as a positive aspect of an individual’s sexual relationship, consisting of stability, satisfaction and a good quality of life [7]. Sexual distress is a condition where individuals have negative emotions and feelings of inadequacy in their sexual relationships. Emotions such as frustration, worry, anxiety and interpersonal difficulty negatively affect overall well-being and quality of life [7].

Women’s sexual health depends on their psychological, emotional, physical and social condition [8]. Therefore, the pandemic may have significantly affected their sexual behavior mainly due to stress and other biological or social factors. Studies showed that the quality of women’s sexual life decreased significantly during the pandemic. This pandemic has been associated with a decreased desire for pregnancy, but increased sexual desire, frequency of intercourse and menstrual disorders [9]. Another study showed a negative influence of the pandemic on sexual function and quality of life of women [10]. There was also an association with sexual dysfunction and decreased libido [11,12].

The aim of this study was to investigate the potential impacts and changes in the quality of women’s sexual lives caused by the measures imposed due to the COVID-19 pandemic, using a systematic review of the literature.

## 2. Materials and Methods

The purpose of this study was to investigate the effect of the COVID-19 pandemic on the quality of reproductive-age women’s sexual life. More specifically, we aimed to compare sexuality parameters, such as sexual functioning, sexual desire and frequency of intercourse, sexual satisfaction and fertility desire, during a period of the COVID-19 pandemic with months prior to said pandemic. This study was not registered in PROSPERO.

### 2.1. Search Strategy

In order to meet this objective, a review of the literature was conducted. Articles were searched for on PubMed/Medline, Google Scholar and Scopus.

### 2.2. Inclusion and Exclusion Criteria

For an article to be included in the review, it needed to be published in English, be primary research (cross-sectional, case-control, cohort), including women of reproductive age (18–50 years), married or cohabiting women (sexually active) or heterosexual couples. The review excluded research involving women who were pregnant, who had another physical condition (sexually transmitted diseases, medication affecting libido, history of infertility) or mental health condition (mental illness, personality disorders, depression) and women who were in sexual minority groups (lesbians, gay, bisexual, transgender).

### 2.3. Study Selection

**Figure 1:**
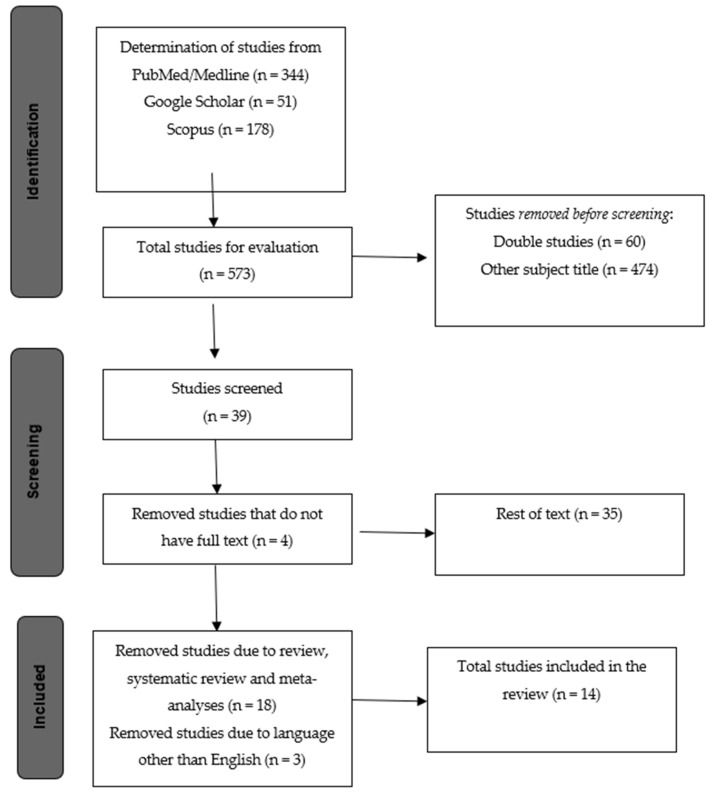
Flow chart: structure search strategy.

Considering that the COVID-19 pandemic started in December of 2019, we set a filter for the date of publication to be during 2020 and 2021. The initial research yielded 573 articles, which were first screened by title and abstract, excluding 538 articles not related to the subject of this paper. Subsequently, the remaining 35 studies were examined against the inclusion and exclusion criteria and 18 were excluded as they were reviews, and another 3 as they were published in languages other than English, leavingonly 14 studies to be included in the review (Figure 1). The study analyzed the effect of the COVID-19 pandemic on the quality of women’s sexual life. Different domains of sexual life were analyzed, such as sexual functioning, sexual desire, frequency of intercourse, sexual satisfaction and fertility desire, compared to before the COVID-19 pandemic, and the role of psychological factors, such as anxiety and depression on this change. More specifically, the research articles identified through the initial review are mentioned first.

Nine criteria were used to evaluate the articles’ methodological quality. Four criteria made up the first group of criteria about the selection process. Eight papers satisfied the first requirement, which was related to the representative exposure sample. Only four studies met the second condition for the selection of non-exposed, which was whether researchers had information about the women’s sexual lives prior to the pandemic to compare with. Seven studies that recorded exposure to COVID-19 met the third criterion regarding the exposure finding. The fourth condition was satisfied by all studies because the outcome did not arrive before the exposure. The changes in female function scores were each study’s final finding. Two criteria about the adjustment for confounding circumstances madeup the second group of criteria (comparability). All articles but two met the sixth criterion, which was modification for educational level. As an adaptation for at least one extra confounding factor that had been used in all research, the sixth criterion was satisfied by all articles. Three criteria made up the third category of criteria (results), which assessed the caliber of each study’s outcomes. The seventh criterion for assessing the sexual lives of women during the pandemic was met by all research studies. There is no need for further investigation because the main objective of the research was to assess how women’s sexual lives were affected by the pandemic. Therefore, all research also satisfied the eighth condition. The final requirement, non-bias, was also met by all articles. The studies’ grades ranged from 6 to 9 (Table 1).

## 3. Results

In this review we included 14 studies which were conducted in various countries, mainly in Europe. All studies had a cross-sectional design, and four of them [9,10,13,14] evaluated the sexual life of a cohort before and during the pandemic (Table 2). Regarding the methodological quality of the studies, only one study was evaluated as having a very good quality [14], while the rest of them were of moderate methodological quality (Table 1).

One of the most common factors reported to affect the sexual life of women during the pandemic was the psychological factor, which was evaluated in seven studies [12,13,15,16,17,18,19,20,21]. Specifically, Bhambhvani et al. [13] assessed the impact of the COVID-19 pandemic on female sexual function and frequency in 91 women from the United States. Evaluating the sexual functioning using the FSFI score, women mentioned a decrease during the pandemic, with a greater decrease found in domains related to arousal, lubrication and satisfaction.

### 3.1. Psychological Factors Associated with Women’s Sexual Life during the COVID-19 Pandemic

The aim of the study of Carvalho et al. [18], which was conducted in Portugal, was to evaluate the relationship between the COVID-19 confinement levels and sexual functioning domains in 662 participants. Based on the data of the 417 women studied, authors observed no relationship between confinement and sexual functioning in women, but they observed a strong effect of psychological factors on women’s sexual functioning. Specifically, they concluded that psychopathological symptomatology could predict the levels of sexual desire, sexual arousal, lubrication, orgasm, sexual satisfaction and sexual pain. An Italian study conducted by Costantini et al. [15] analyzed changes in couples’ sex lives during the COVID-19 lockdown. In this research, 2151 participants (1112 women, 1039 men) were included. Most of the responders (n = 579) answered that their sexual life improved, 314 that it deteriorated, while 219 reported no change. Women who responded that their sex lives deteriorated presented no sexual dysfunction, but they had increased rates of anxiety, tension, fear and insomnia. Other factors associated with the worsening of sexual life were being unemployed or smart working or having sons. Szuster et al. [12] conducted a survey to investigate the impact of the pandemic on the mental, physicaland sexual health of 1644 Polish females. The frequency of sexual activity and libido level significantly decreased after the pandemic breakout, with sexual functioning being strongly correlated with depression. The sexual functioning was also associated negatively with the presence of any comorbid chronic disease, the intensity of the fear of infection and fear of health conditions, perceived loneliness and being up to date with media news. Omar et al. [20] investigated the sexual satisfaction of 773 married Egyptians (484 women, 289 men) during the pandemic. As expected, sexual satisfaction decreased during the lockdown. A huge proportion (97.3%) of female participants mentioned sexual dysfunction. Sexual relation stress was higher in housewives and unemployed women, women whose husbands were >35 years old, who were married for 5–10 years, and who had higher rates of anxiety and sexual dysfunction. The sexual function and relationship quality of couples during the lockdown was investigated in the study conducted in Greece by Sotiropoulou et al. [19]. The study included 299 adult heterosexual participants in a relationship (213 women, 86 men). Surprisingly, little or no negative impact on sexual function was reported. The lockdown increased anxiety and negatively affected mood only in cases where partners could not get in touch. Satisfaction by sexual activity and emotional security was reported by those being in a steady relationship and living with their partner. De Rose et al. [21] conducted research in order to investigate the effect of depression on sexual activity in a sample of hospital workers and their acquaintances during the COVID-19 lockdown in Italy. The study included 544 participants, of which 284 were females and 260 were males. The sexual desire was normal in 57.7% of women responders and low in the rest, while the sexual satisfaction was low in 86.6%. Low sexual desire was significantly associated with age and sexual satisfaction, but no correlation with depression was observed.

### 3.2. Other Factors Associated with Women’s Sexual Life during the COVID-19 Pandemic

Other factors were also reported to affect the sexual life of women during the COVID-19 lockdown. Schiavi et al. [10] included in their study 89 Italian non-infected reproductive-age women, living with their sexual partner, and assessed the impact of the pandemic on their sexual function and quality of life. Results showed a significant reduction in mean sexual intercourse/month, sexual functioning and quality of life, while sexual distress increased. Lower sexual functioning was associated with working outside the home, university educational level and parity. In addition, Karagöz et al. [17] investigated the effect of the COVID-19 pandemic on Turkish couples’ sexuality. The study included 108 women and 162 men. Results showed an important decrease in sexual functioning scores and sexual intercourse compared to the pre-pandemic period, while sexual avoidance and solitary sexual approach behaviors (masturbation or watching sexual content videos, etc.) increased. A very important finding was that women who spent more time with their spouse had better sexual function. A corresponding study conducted by Kovalak et al. [16] found that among 169 Turkish women, the sexual desire was not affected in 58.6% of them but was decreased in 32.9%. Sexual desire demonstrated a greater decrease in women whose income decreased during the pandemic, than in women with a stable income. Interestingly, the sexual satisfaction increased more in women with decreased income. Culha et al. [22] investigated changes in the sexual lives of Turkish health professionals due to the COVID-19 outbreak. Eighty-nine women and ninety-six men answered the online survey investigating sexual functioning, anxiety and depression. Results showed a decrease in sexual desire, weekly sexual intercourse/masturbation number, foreplay time and sexual intercourse time, compared to before the COVID-19 outbreak. It is interesting that during the lockdown, health care workers preferred less foreplay, oral sex and anal sex and more non-face to face sexual intercourse positions. The only factor associated with sexual dysfunction in women was alcohol drinking. The study of Neto et al. [11] evaluated the impact of the pandemic on sexual functioning in 1314 healthcare professionals and medical students (928 women, 386 men) at a reference center for the treatment of COVID-19 in Brazil. The sexual satisfaction decreased in almost half of the participants. The decrease was associated significantly with lower libido, missing nightlife, higher masturbatory frequency and isolation from the partner. Worsening of libido was also observed in a significant proportion of participants and was associated with missing of nightlife, older age and isolation from the partner. Participants who did not report worsening of sexual function were those who remained sexually active during the pandemic and had a higher sexual frequency. Fuchs et al. [14] compared the sexual health of 764 women in Poland before and after the COVID-19 pandemic outbreak. Comparing the sexual function before and during the pandemic, researchers observed a significant decrease, which was statistically significant for every domain (desire, arousal, lubrication, orgasm, satisfaction and pain). Sexual function influence depended on the workplace, with a bigger worsening observed for women who did not work at all. The Turkish women’s sexual behavior before and during the pandemic was investigated by Yuksel and Ozgor [9]. The average frequency of sexual intercourse significantly increased during the pandemic compared with the pre-pandemic period, although sexual functioning worsened. Regarding the desire to become pregnant, fewer participants responded positively during the pandemic, but contrariwise, use of contraception significantly decreased. Moreover, menstrual disorders became more common during the pandemic than before (Table 3).

## 4. Discussion

The aim of this study was to investigate the potential impacts on and changes in the quality of women’s sexual life caused by the measures imposed due to the COVID-19 pandemic. The results of the review provide strong evidence that the sexual life of women has been significantly affected by the pandemic, demonstrating a great worsening in various domains. This effect seems to be mediated mostly by psychological factors, such as depression [12,18,20] and anxiety [15,18,20]. Moreover, women who did not work during the pandemic showed a greater worsening of sexual function [10,20].

Most studies reported a great decrease in sexual functioning during the pandemic [9,10,14,17,18,20], but most participants in Sotiropoulou et al.’s study [19] mentioned little or no impact. Sexual satisfaction was also reported to be significantly decreased in all studies where it was investigated [11,13,20,21], as well as the libido level [12]. Sexual distress was also increased during the COVID-19 pandemic [10] and a lot of people showed sexual avoidance and solitary sexual approach behaviors [17].

The impact of the pandemic on other aspects of sexual life is still controversial. Despite the expected, Costantini et al. [15] mentioned that most couples’ sex lives improved during the pandemic. Two studies [16,21] reported normal sexual desire during the pandemic, while one [22] observed a significant decrease. Sexual frequency is another measure that was affected in multiple ways. Four studies suggest that sexual frequency is negatively affected during the lockdown [10,12,17,22], Bhambhvani et al. [13] mentioned no change, while Yuksel and Ozgor [9] showed an increase. The desire to become pregnant was investigated by Yuksel and Ozgor [9] and seemed to be decreased during the pandemic. Corresponding results are reported in the study by Naya et al. [23], where most participants indicated that they would postpone their desire to start a family until later due to the pandemic. However, interestingly, the use of contraception decreased significantly during the lockdown period [10] This is consistent with findings from other studies reporting a decrease in contraceptive use [24,25]. In these studies, researchers attribute this decrease to the limitation of personal contact, whichmakes contraception useless anyway [24], or the inability to access contraceptive methods, either due to the lockdown or due to economic hardships brought on by the pandemic [25].

The most important factor affecting women’s sexual life that emerged from this study was the psychological factor. A lot of studies have been published regarding the psychological impact of COVID-19 [26,27] and psychology plays a major role in sexual desire [28,29] and activity [30,31], since good psychological mood increases sexual desire, so this finding was expected. Interestingly, neither the confinement [18] nor the infection status [11,14] was correlated with women’s sexual life. Evaluating only the psychopathological symptomatology of women was enough to predict the levels of sexual desire, sexual arousal, lubrication, orgasm, sexual satisfaction and sexual pain [18]. Even Costantini et al. [15], who mention an improvement in sexual life during the pandemic, also mention that women with worse sexual lives are typically those with high levels of anxiety, tension, fear and insomnia. Depression was significantly correlated with sexual functioning [12], but not sexual desire [21].

Another commonly reported factor affecting women’s sexual life is working status. Out-of-home work was correlated with decreased sexual functioning [10]. People working outside the home are in more danger of being infected, so it is normal for them to be afraid of having sex to not infect their partner [32]. This is in line with the findings of Szuster et al. [12] who observed a worsened sexual functioning in women who had a higher intensity of fear of infection. Additionally, unemployed women reported worse sexual functioning [14] and satisfaction [20]. Women who are not working at all are totally economically dependent on their husbands, and so they are more affected by their partner’s stress due to the economic insecurity that has arisen with the pandemic. Regarding women who lost their jobs during the pandemic, unemployment is surely connected with additional stress and fear.

### Strengths and Limitations

This is the first systematic review that investigates the effect of the COVID-19 pandemic on women’s sexual life. In this article, we investigate all the factors that have been recorded to influence women’s sexual function during the pandemic. Limitations of this study included that articles have been conducted in only a few countries around the world and most studies have a risk of recall bias resulting from the lack of pre-COVID data compared to post-COVID data. Another limitation is the small number of studies on the quality of women’s sexual life, but considering that the pandemic only occurred in the last two and a half years, this is expected. Furthermore, there were also difficulties in comparing survey results on the specific pandemic situation in each country, and difficulties in comparing numerically different sample sizes.

## 5. Conclusions

The COVID-19 pandemic has affected all aspects of people’s lives, either physically or through mental health. Sexual life is one of the most important domains for women of reproductive age. Unfortunately, most of the domains of sexual life have been affected negatively by the pandemic, while for others the exact effects remain unidentified. Restriction measures implemented during the pandemic had significant effects on people’s psychology, resulting in increased stress and depression levels. Previous research has shown a strong correlation between psychology and sexual life. Similar findings arise from this systematic review, as mental health seems to be the main factor affecting sexual life during the pandemic, as the increase in depression and anxiety caused by the pandemic resulted in the worsening of women’s sexual lives. Another important factor that seems to affect women’s sexual life during the pandemic is the working status, with unemployed women or women staying at home showing lower sexual functioning and satisfaction.

Sexual life is one of the most important aspects of an adult’s life. The pandemic has negatively affected the sexual life of a lot of women worldwide. The findings of this study highlight the importance of screening and developing interventions for sexual functioning-related problems, especially in at-risk populations, such as in those facing depression and anxiety because of the pandemic. Additionally, the study shows that women’s sexual lives before the pandemic were much better than during it, so it would be very interesting to investigate whether women whose sexual lives were affected will return back to normal after the end of the pandemic.

## Figures and Tables

**Table 1:** The terms were used.

Component 1		Component 2		Component 3
Female OR Women NOT Pregnant	AND	COVID-19 OR SARS-CoV-2 OR Coronavirus OR COVID-19 pandemic	AND	Sexual function OR Sexual behavior OR Sexual health NOT HIV NOT AIDS NOT Violence

**Table 2:** Methodological Quality of the included articles.

Author/Year	Selection 1 2 3 4	Comparability 5 6	Result 7 8 9	Total
1. Szuster (2021) [12]	* - * *	**	***	8
2. Costantini (2021) [15]	- - - *	**	***	6
3. Kovalak (2021) [16]	* - - *	**	***	7
4. Karagöz (2020) [17]	* - - *	**	***	7
5. Neto (2021) [11]	- - * *	- *	***	6
6. Bhambhvani (2021) [13]	- * * *	- *	***	7
7. Carvalho (2021) [18]	- - * *	**	***	7
8. Sotiropoulou (2021) [19]	- - * *	**	***	7
9. Omar (2021) [20]	* - * *	**	***	8
10. De Rose (2021) [21]	- - - *	**	***	6
11. Culha (2021) [22]	* - - *	**	***	7
12. Fuchs (2020) [14]	* * * *	**	***	9
13. Schiavi (2020) [10]	* * - *	**	***	8
14. Yuksel (2020) [9]	* * - *	**	***	8

Notes: The 9 criteria were 1. Representative exposure sample, 2. selection of non-exposed, 3. exposure finding, 4. outcome did not precede the study, 5. adaptation for educational level, 6. adaptation for additional confounding factor, 7. outcome evaluation, 8. adequate monitoring time, 9. non-bias of wear. The symbol (*) means that the study met the specific criterion and the symbol (-) means that the study did not meet it. Selection has 4 criteria 1. 2. 3. 4. Comparability has 2 criteria 5. 6. Results has 3 criteria 7. 8. 9.

**Table 3:** Studies included in the systematic review.

Author/Year/Design/Start-Expiry	N	Data	Country	Exposure	Measures	Outcomes	Additional Outcomes	Additional Risk Factors
1. Szuster (2021) [12] Cross-sectional study 22 April–7 May 2020	1644 women	Online survey from Medical University, Wrocław, Poland.	Poland	COVID-19 pandemic	Sexual function: FSFI Depression: BDI	Low sexual functioning.	High levels of depression, intensity of the fear of infection and fear of health conditions, perceived loneliness and being up to date with media news.	Comorbid chronic disorders
2. Costantini (2021) [15] Cross-sectional study 4–18 May 2020	1112 women, 1037 men	Urological centers	Italy	COVID-19 pandemic	Sexual function: FSFI Couples’ relationships: MAT Anxiety: HAM Effects of the COVID-19 pandemic and quarantine on couples’ relationships: in-house questions	Normal sexual functioning.	Anxiety, tension, fear and insomnia	Having sons, being unemployed or smart working.
3. Kovalak (2021) [16] Cross-sectional study 27 June–July, 2020	169 women	İstanbul Bağcılar Training and Research Hospital	Turkey	COVID-19 pandemic	Sexual life during the COVID-19 pandemic: questionnaire based on previous studies	Low sexual desire. Higher sexual satisfaction.	Decrease in income	Lower income level
4. Karagöz (2020) [17] Cross-sectional study 6–20 May 2020	108 women, 162 men	Bursa YuksekIhtisas Training & Research Hospital and online survey	Turkey	COVID-19 pandemic	Sexual function: FSFI Anxiety: GAD-7 Depression: PHQ-9 Subjective stress perception: PSS	Sexual intercourse and sexual functioning decreased, sexual avoidance and solitary sexual approach behaviors increased.		
5. Neto (2021) [11] Cross-sectional study 22 July–24 August 2020	928 women, 386 men	Hospital das Clinicas—University of Sao Paulo Medical School	Brazil	COVID-19 pandemic	Sexual function: FSQ Effects of the COVID-19 pandemic and quarantine on couples’ relationships: in-house questions	Worsening of sexual satisfaction, lower libido.	Missing nightlife, isolation from the partner, lower libido, higher masturbatory Frequency	Older age.
6. Bhambhvani (2021) [13] Cross-sectional cohort study 20 October–1 March 2020	91 women	Partner cannabis dispensary	United States	COVID-19 pandemic	Sexual function: FSFI Anxiety and depression symptoms: PHQ-4	No change in sexual frequency. Low sexual functioning.	Anxiety and depression symptoms.	
7. Carvalho (2021) [18] Cross-sectional study 19 March–1 June 2020	417 women, 245 men	Online survey	Portugal	COVID-19 pandemic	Sexual function: FSFI Psychological adjustment during confinement: BSI	Low sexual functioning.	Psychological adjustment.	
8. Sotiropoulou (2021) [19] Cross-sectional study 21 April–3 May 2020	213 women, 86 men	Online survey	Greece	COVID-19 pandemic	Sexual function: FSFI Sexual activity: in-house questions Relationship quality: in-house questions Mood and anxiety: in-house questions	Satisfaction by sexual activity and emotional security.	Increased anxiety and deficient mood.	Couples living with their children.
9. Omar (2021) [20] Cross-sectional study March–27 June 2020	484 women, 289 men	Online survey	Egypt	COVID-19 pandemic	Sexual function: FSFI Sexual satisfaction: ISS Anxiety: GAD-7 Depression: PHQ-9	Low sexual performance satisfaction, low sexual functioning, high sexual stress.	Anxiety.	Being a housewife or unemployed, husband’s age >35 years, marriage duration of 5–10 years, female sexual dysfunction.
10. De Rose (2021) [21] Cross-sectional study 8 April–2 May 2020	284 women, 260 men	Policlinico San Martino Hospital and Online survey	Italy	COVID-19 pandemic	Sexual function: FSFI	Low sexual desire.		Being a health care worker, having children at home, being female, living with the partner
11. Culha (2021) [22] Cross-sectional study 2 May–26 May 2020	89 women, 96 men	Cemil Tascioglu City Hospital, Istanbul and Online survey	Turkey	COVID-19 pandemic	Sexual function: FSFI Anxiety: STAI-1, STAI-2 Depression: BECK	Low sexual desire, weekly sexual intercourse/masturbation number, foreplay time and sexual intercourse time. Less foreplay, oral and anal sex and more non-face to face sexual intercourse positions reported.		
12. Fuchs (2020) [14] Cross-sectional cohort study March–April 2020	764 women	Medical University of Silesia	Poland	COVID-19 pandemic	Sexual function: FSFI Effects of the COVID-19 pandemic and quarantine on deterioration of material standing, workplace change and the frequency of sexual intercourse: in-house questions Levels of stress and anxiety: in-house questions	Lower sexual functioning.		The workplace or not working
13. Schiavi (2020) [10] Cross-sectional cohort study February 2018–February 2020	89 women	Sandro Pertini Hospital Rome, Campus Biomedico University Rome	Italy	COVID-19 pandemic	Sexual function: FSFI, FSDS Quality of life: SF-36	Low sexual functioning. Quality of life and mean sexual intercourse /month decreased, sexual distress increased.		Parity ≥1.
14. Yuksel (2020) [9] Cross-sectional cohort study 1 February 2018–30 September 2019 and pandemic period (11 March–12 April 2020)	58 women	Esenler Maternity and Children’s Hospital Instanbul, Haseki Teaching and Research Hospital Instanbul	Turkey	COVID-19 pandemic	Sexual function: FSFI Effects of the COVID-19 pandemic on frequency of sexual intercourse, desire for pregnancy, contraception type and presence of vaginal infection: in-house questions	Sexual intercourse and menstrual disorders increased, Sexual functioning, desire to become pregnant and use of contraception decreased.		

Notes: BDI: Beck Depression Inventory (depression). BSI: Brief Symptom Inventory (psychological adjustment). FSDS: Female Sexual Distress Scale (Sexual distress). FSFI: Female Sexual Function Index (sexual functioning of women). FSQ: Female Sexual Quotient (sexual functioning of women). GAD: Generalized Anxiety Disorder (anxiety). HAM-A: Hamilton Anxiety Rating Scale (anxiety). ISS: Index of Sexual Satisfaction (sexual satisfaction). MAT: Marital Adjustment Test (couples’ relationships). PHQ: Patient Health Questionnaire (depression). PSS: Perceived Stress Scale (anxiety). SF-36: 36-Item Short Form Survey (quality of life). STAI: State-Trait Anxiety Inventory (anxiety).

## Data Availability

Not applicable.
